# Providing Outpatient Oncology Mental Health Support: Understanding Staff Perspectives

**DOI:** 10.1002/pon.70206

**Published:** 2025-06-13

**Authors:** Elizabeth Matthews, Kate Webber, Joshua F. Wiley, Catriona Parker

**Affiliations:** ^1^ Faculty of Medicine Nursing and Health Sciences School of Psychological Sciences Monash University Clayton Australia; ^2^ Oncology Department Monash Health Clayton Australia; ^3^ School of Clinical Sciences Monash University Clayton Australia; ^4^ School of Public Health and Preventive Medicine Monash University Clayton Australia; ^5^ Supportive and Palliative Care Unit Monash Health Clayton Australia

**Keywords:** attitude of health personnel, cancer, distress, focus groups, health resources, oncology, psycho‐oncology

## Abstract

**Objective:**

Mental health challenges are common in individuals with cancer, but accessing support remains a gap, particularly in outpatient oncology. Real‐world factors influencing staff's ability to integrate mental health assessment and support into workflows are unclear. We aimed to (1) identify and explore factors influencing the assessment and management of mental health in outpatient oncology and (2) identify factors that influence normalising support for mental health during cancer treatment.

**Methods:**

Through an exploratory qualitative approach, data was collected via focus groups with oncology healthcare professionals at a major metropolitan hospital. Reflexive thematic analysis revealed seven themes.

**Results:**

Staff recognised the critical need for mental health support during treatment. However, staff participation in mental health assessment and referral processes was impeded by feelings of futility due to the lack of available oncology‐specific services. Staff reported insufficient resources and time constraints, and reliance on experiential knowledge rather than the preferred support from clinicians with mental health training. Staff highlighted inequities in access to mental health support which exacerbate disparities in care provision. Staff reported that a lack of role clarity and responsibilities hindered monitoring of patient mental health management and contributed to a perceived lack of accountability. Short‐term initiatives such as improved documentation, creation of central referral pathways and clinical supervision for all staff were reported as desirable.

**Conclusions:**

While systemic resource constraints were acknowledged, oncology staff also identified several feasible, shorter‐term initiatives they felt would be helpful and desirable until mental health professionals can be integrated into oncology outpatient care.

## 1 Background

Distress is an expected reaction to a cancer diagnosis. Unmanaged or ineffectively managed distress can lead to worse patient outcomes including lower quality of life [1, 2], reduced treatment adherence [3], more frequent hospital stays [4], higher healthcare costs [5] and ultimately increased mortality rates [6]. Compared to other sources of distress (e.g. from physical side effects of treatment), psychological distress and resultant mental health concerns in people with cancer contribute disproportionately to overall distress levels [7, 8]. Common manifestations of psychological distress in cancer include worry, sadness, fear, or anger, and while often transient, these symptoms can develop into mental health conditions such as depression or anxiety that persist beyond cancer treatment [9, 10]. Examples of interventions to target these concerns include supportive counselling, cognitive behavioural therapies, and integrated consultation models [11].

Adequate management of mental health in people receiving cancer treatment remains an unmet need [12, 13]. Routine screening for psychological distress and mental health symptoms is recommended as standard care in Australia [14]. However, implementation into standard practice is not well described and appears inconsistent [15]. Internationally, integrated psycho‐oncology models where care is shared between oncology, primary care, and mental health professionals have demonstrated improvements in patient outcomes, including reduced distress, better mental health symptom management, and enhanced quality of life [16, 17, 18].

The factors that influence oncology staff's ability to implement guidelines into their workflow, and those that prevent the full integration of holistic mental health monitoring and management into oncology, remain unclear.

Research has documented oncology specific challenges that influence the integration of mental health assessment and referral, including: lack of time for distress screening [15, 19], lack of training [15, 19], a perception that mental health management is lower priority to people with cancer [20], and a lack of resources to action positive distress screens (e.g., referrals, on‐site treatment) [15]. Most Australian healthcare professionals (HCPs) report rating psychological support services as beneficial to their patients, yet most patients are not referred [15]. Whether, how and the extent to which these and other challenges manifest and influence healthcare in Australia's oncology system has received relatively little research. Understanding HCPs' perspectives via qualitative methods will broaden the applicability of research findings and inform directions for improvements to how mental health is being addressed within outpatient oncology.

We aimed to (1) identify and explore factors influencing the assessment and management of mental health in outpatient oncology and (2) identify factors that influence normalising support for mental health during cancer treatment.

## 2 Methods

Monash Health Human Research Ethics Committee reviewed and approved this project (Approval number: RES‐22‐0000‐521A). A descriptive qualitative approach was chosen to facilitate the exploration of HCPs' experiences managing patient mental health. Reporting follows COREQ guidelines [21] with details provided in Supplementary Materials.

### 2.1 Participants

Purposive and snowball sampling techniques via pre‐existing hospital staff networks were used for recruitment and to ensure a broad range of perspectives and experiences were included. Inclusion criteria were that participants needed to be currently working in a patient‐facing role within an outpatient oncology team at a specific metropolitan hospital. There were no exclusion criteria. Informed consent was obtained from all participants included in the study.

### 2.2 Data Collection

Focus groups were chosen to allow individual perspectives and experiences to be collected while also allowing for ad hoc interaction, a shared understanding and troubleshooting to occur between group members. Groups were run online between November 2023 and April 2024. A focus group guide was developed based on the wider literature to assist in exploring HCPs' experiences managing patient mental health within the current system. The exploratory questions used in the focus groups are in Table 1. Additional prompting was used to clarify content and encourage in‐depth topic exploration. Data collection ceased once data saturation was achieved, defined as the point at which no new information surfaced [22]. Focus groups were audio recorded and transcribed verbatim before being de‐identified, transcripts were then uploaded to *NVivo* (Version 14.23.4, QSR International) to facilitate analysis.

**TABLE 1:** Focus group guide.

– Tell me about how patient mental health is currently being managed during the treatment period. – What is working well about how you manage patients who are struggling with their mental health while being treated? – What are the challenges with supporting the mental health of your patients? – What do you think could be done better to manage psychologically distressed patients?

### 2.3 Data Analysis

Reflexive thematic analysis [23] was utilised to identify thematic patterns across the dataset. Both inductive and deductive coding approaches were employed to identify key themes in the transcripts. Open codes were generated from initial concepts identified in the focus group transcripts. After coding all the transcripts, initial focus groups were inductively reanalysed at the end of data collection to ensure no coding was missed in earlier transcripts. Axial codes were then developed to link these concepts and connect codes before themes were defined and named. Frequent discussions among the research team about data helped to ensure the robustness of data interpretation. The research team included members with backgrounds in psycho‐oncology, qualitative methods, and health psychology, with further details on team member backgrounds and data coding provided in Supplementary Materials. Preliminary results were presented back to departmental stakeholders during data analysis with opportunity for feedback provided.

Themes were then mapped to the constructs of Normalisation process theory (NPT). NPT seeks to assist with understanding the practices contributing to the integration of new interventions into the healthcare system to the extent that they become routine [24]. NPT seeks to take into account the complexity of real‐world healthcare settings and local working practices to allow for identification of opportunities for service improvement that are feasible and practical to stakeholders in the process [25]. NPT has been used to assist in understanding factors influencing integration of complex interventions and processes into health care settings [26], and uses four constructs to assist with assessment, detailed in Table 2.

**TABLE 2:** NPT construct definitions [27].

NPT construct	
Coherence	Sense‐making by participants, the understanding individuals and organisations need to facilitate embedding of a practice into routine care
Cognitive participation	Relates to the work individuals and organisations need to do to engage with a process and the willingness of individuals to engage with a process
Collective action	Refers to the work required by the individual and the organisation to complete the process and how this can fit in with current processes
Reflexive monitoring	How individuals or the organisation appraises and assesses the effectiveness of the process

## 3 Results

### 3.1 Participants

The sample comprised 26 participants across 8 focus groups (run time *Mean* = 58 min; range 55–62 min, group size 2—5 participants) currently working in outpatient oncology. Ten participants were medical staff, 15 were nursing staff and one was an Allied Health staff. Job roles have been grouped for anonymity, participants included senior medical oncologists, junior medical staff, nurse practitioners, registered nurses and cancer support nurses. Participants had between 11 months and 34 years of outpatient oncology experience (*Mean* = 15 years), with seven participants having over 20 years of experience in oncology and five having less than 5 years of experience.

Seven themes were identified and mapped to NPT constructs (see Table 3) to contextualise the content of the theme and its influence on the overall normalisation of mental healthcare within the oncology outpatient setting. Each theme is discussed in the following sections.

**TABLE 3:** NPT constructs [27] and corresponding themes.

NPT construct and it's description		Theme
Coherence	Sense‐making by participants, the understanding individuals and organisations need to facilitate embedding of a practice into routine care	1. Mental health support during treatment is an unmet need that impacts medical care
Cognitive participation	Relates to the work individuals and organisations need to do to engage with a process and the willingness of individuals to engage with a process	2. Barriers to initiating conversations about mental health 3. A desire for integrated mental health care within oncology
Collective action	Refers to the work required by the individual and the organisation to complete the process and how this can fit in with current processes	4. Reliance on experience in the absence of formal mental health supports 5. A lack of capacity to prioritise mental health support 6. Available support is inequitable
Reflexive monitoring	How individuals or the organisation appraises and assesses the effectiveness of the process	7. Lack of accountability around managing patient mental health

#### 3.1.1 Coherence: Sense‐Making by Participants, the Understanding Individuals and Organisations Need to Facilitate Embedding of a Practice Into Routine Care

##### 3.1.1.1 Theme 1: Mental Health Support During Treatment is an Unmet Need That Impacts Medical Care

HCPs consistently identified that mental health during treatment is ‘*something we need to address as clinicians more’* (P8) and were unanimous in their view that current management is insufficient. Clinicians explained that for some patients, the inability to support and manage mental health during treatment led to instances where ‘*life span was shortened as a result’* (P22) of suboptimal treatment engagement. HCPs felt strongly that psychological support during treatment is required as part of cancer treatment as an adjunct to medical treatment: *‘we have to have a chemotherapy nurse because if you don't deliver chemotherapy you are not doing the basics of cancer care…equally we should have… mental health care professionals’* (P4). A clear distinction was drawn between patients who have pre‐existing mental health conditions who are *‘actually well supported’* (P19), and those whose mental health distress is directly linked to ‘*not coping with the whole experience of having cancer’* (P9). The strongest unmet needs were identified to be related to ‘*the psychosocial and existential angst that patients have’* (P9) and needing *‘help adjusting to their diagnosis’* (P5). Cancer‐specific mental health support services ‘*that can respond in a timely and comprehensive manner’* (P5) were repeatedly noted as strongly desired, but absent from current care pathways, something that was reported as noticeable to patients.

Nobody really has access to ongoing cancer‐related psychological support … that just doesn't exist…patients kind of expect that it does exist and are … taken aback when it doesn’t.

(P4)

#### 3.1.2 Cognitive Participation: Relates to the Work Individuals and Organisations Need to do to Engage With a Process and the Willingness of Individuals to Engage With a Process

##### 3.1.2.1 Theme 2: Barriers to Initiating Conversations About Mental Health

Due to a lack of adequate referral and management options, HCPs disclosed that mental health had become *‘something that you actively avoid asking about’* (P9). Personal discomfort around addressing patient mental health was also reported as a factor in initiating conversations about mental health: *‘it's hard on the nurses … to have those conversations’* (P20). HCP mood was also reported as a deterrent to cognitive participation in the assessment and referral process for patient mental health: *‘my mood and the sort of the stress of today contributes to my willingness to want to really engage or not really engage’* (P12). HCPs expressed that feelings of futility asking about mental health and avoidance of asking, stem from an inability to refer to appropriate support: *‘every clinic somebody is whinging to me about their mental health and I have nothing to offer them and it drives me nuts’* (P6), *‘you feel really helpless as the health professional when they're asking for help and there's not much you can provide for them’* (P23).

##### 3.1.2.2 Theme 3: A Desire for Integrated Mental Health Care Within Oncology

HCPs repeatedly expressed a desire for mental health care to be *‘integrated in [to] our service’* (P5) in a meaningful way. Examples of meaningful integration included having *‘a psychologist or a psychiatrist who can actually be there and be a part of the team rather than this person on the bottom of a letterhead’* (P9), where mental health professionals are ‘*part of the multi‐disciplinary team*’ (P6) and *‘more social workers here [in the chemotherapy day unit]’* (P17). As well as providing support directly to patients, HCPs wanted mental health professionals accessible to them: *‘It would be really useful to have…somebody we can talk to easily about patients we're worried about’* (P1), *‘I'd also like to see support for oncology staff… clinical supervision being made accessible routinely as well to help with staff mental health and dealing with very complex patients’* (P22).

#### 3.1.3 Collective Action: Refers to the Work Required by the Individual and the Organisation to Complete the Process and How This can Fit in With Current Processes

##### 3.1.3.1 Theme 4: Reliance on Experience in the Absence of Formal Mental Health Supports

The ‘*lack of understanding of mental health conditions’* (P1) and a lack of ‘*any formal mental health training … since med[ical] school’* (P9) has led HCPs to rely on experiential learning rather than formal training. Decision‐making is guided by ‘*what you've learnt from necessity and just by example’* (P15):

We get things done because…we just knew who the right people were to go and talk to and ask.

(P14)

Current management relies on a few key staff members who ‘*stop everything from falling apart*’ (P12) and who have ‘*very, very good institutional knowledge having been here for a number of years’* (P7). HCPs felt that the addition of staff with specialised mental health knowledge would improve their ability to address patient mental health with patient navigators, nurse practitioners, or social workers and psychologists *‘with expertise in oncology’* (P11) all viewed as potential knowledge sources.

##### 3.1.3.2 Theme 5: Limited Time to Prioritise Mental Health Support

HCPs reported insufficient time to provide integrated mental health support during treatment:

I know I haven't got the time to invest in what they need because I know I don't have the time to invest what I have in what I have to do.

(P14)

HCPs reported needing to limit conversations about mental health to prioritise physical health concerns due to lack of time to provide holistic care: ‘*you've got a 20* min *appointment…I struggle to do all the medical stuff in that time’* (P4). Participants identified that often ‘*we know that somebody might need something, but we don't necessarily have the time to invest in it’* (P14) with the lack of ‘*private space for talking’* (P22) for nursing staff further preventing conversations about mental health from occurring. The department's capacity to consistently support patient mental health and engage in sustained collective action is challenged by these constraints, often placing the burden of service identification back on patients, whose access to support often then depends on *‘how resourceful the patient is’* (P26).

##### 3.1.3.3 Theme 6: Available Support is Inequitable

Participants reported that *‘inequity of care is a real challenge’* (P2), and that *‘there is a disparity without a doubt’* (P26) in what patients are currently able to access. For tumour groups where there was more resourcing and support available this was reported to be visible to patients:

A quote from one of my patients is ‘Why is it that the breast patient gets a nurse to come and talk to her, and I've got bowel cancer and there's nobody for me to talk to?’.

(P1)

Cultural and language barriers further contribute to disparities: *‘It's hard enough to find* *support when you're white and speak English’* (P9), with feelings that *‘public health should really be able to* *support those patients in terms of getting the psychological* *support as well’* (P11). For those wishing to access support in the community *‘the out of pocket cost can be prohibitive for many of the patients’* (P13). HCPs expressed a desire that any changes to current processes be equitable. HCPs from tumour streams with higher resourcing felt that *‘what we have is really what should be the bare minimum for everybody’* (P9). When describing ideal changes to current practices, there was a clear collective commitment to advocating for systemic changes that promote fairness and inclusivity of care.

#### 3.1.4 Reflexive Monitoring: How individuals or the Organisation Appraises and Assesses the Effectiveness of the Process

##### 3.1.4.1 Theme 7: Role Clarity and Accountability for Managing Patient Mental Health

HCPs highlighted a need for staff to have clarity regarding whose role includes managing mental health assessment and referral. In the absence of accessible mental health professionals, there is a lack of clarity about job roles and responsibility in relation to mental health. Some HCPs expressed attitudes that ‘*we don't have the expertise ourselves’* (P22) and therefore ‘*its really not our role*’ (P14). This attitude is compounded by a lack of clarity around referral pathways and whose job it is to refer for onward support:

…I'm too busy and I'll leave it to [ward staff], but they're too busy and they've all left it to [me]…or we think the GP is going to, the consultant's going to do it, and they don't do it.

(P14)

The absence of specific staff delegated to monitor patient mental health management was described as a contributor to inaction: *‘I would guess that that's quite a big component of it…the lack of accountability. So if you ask me, how do I feel about [assessment and referral] not happening? Well, I don't really feel like a huge deal because like I don't actually know the scale of that issue’* (P8). The absence of clear reflexive monitoring means the scale of mental health management needs is unknown, and this ambiguity paired with uncertainty on whose role it is to manage mental health undermines concrete steps for sustained improvements in mental health management.

#### 3.1.5 Short‐Term Opportunities to Enhance Mental Health Support in Outpatient Oncology

Despite the challenges noted, HCPs felt that short‐term initiatives were both beneficial and feasible. HCPs suggested that refining the way mental health is documented in clinical notes would help clinicians to know if the patient has discussed their mental health as part of another visit: ‘*it's not always easy to find out if someone has [discussed it]’* (P16). A reduction of ‘*vague aphorisms like struggling with illness where you don't know if that's a physical or emotional problem’* (P8) and ensuring any discussion about mental health are explicitly referenced so other staff don't need to ‘*spend hours trawling through [medical records]’* (P16) would assist to target limited clinical time to mental health where helpful.

Similarly, collation of experiential and institutional knowledge into centralised and accessible referral pathways with ‘*a very rigid structure*’ (P14) to make it ‘*clearer whose responsibility is it’* (P14) would assist to ensure mental health is supported in a more ‘*formalised and automatic*’ (P9) way. HCPs have the motivation and desire to refer on but lack the time to work out how to do this: ‘*I don't necessarily know what's available…going through intermediates takes longer*’ (P8).

HCPs strongly desired ‘*support for oncology staff…with clinical [mental health] supervision made accessible*’ (P22) to assist with having open conversations around mental health—‘*it's really, really important to be able to chat things through with each other*’ (P25). As well as peer‐based supervision, HCPs also felt strongly that supportive care focussed multidisciplinary meetings could provide a forum to troubleshoot as well as potentially being a training support for junior staff.

## 4 Discussions

This study investigated the factors impacting mental health management during outpatient oncology treatment. We identified multiple factors influencing the effectiveness of current practices and contributing to a lack of normalisation and integration into standard clinical practice. Like previous research, lack of time to speak to a patient about their mental health or to refer patients to mental health support [15], as well as a lack of staff with formal mental health training [19] were contributors. Additionally, this study documented feelings of futility and lack of accountability to further contribute to HCPs ability and willingness to engage patients in conversations about their mental health.

HCPs in this study expressed a desire for clearer and more accessible pathways to manage patient mental health during outpatient treatment. Clearly defined mental health care goals tailored to specific institutions, their resources and their patient population are crucial for sustained implementation—it is important to meet staff expectations that the supports that they can offer to patients will be accessible and will not incur out‐of‐pocket costs in areas where this would be unsustainable for many patients. Establishing institutional standards may help to improve organisational accountability and help to start integrating mental health care more formally into oncology staff workflows, enhancing patient engagement with treatment.

Clarification of roles and responsibilities may also help to manage feelings of futility expressed by HCPs in this study—for example oncology staff who perceive themselves to have more control over their patient care are at lower risk for feelings of guilt and helplessness [28]. Providing clear role boundaries and expectations that staff can follow may assist to reduce feelings of futility by giving clinicians guidance on what supports they may be able to offer to their patients even where integrated or sustained private psychological support may be inaccessible. As suggested by participants in this study, peer support and discussion of staff experiences providing mental health support to patients may also provide opportunities to alleviate feelings of futility. Peer support within oncology is cost‐effective and reduces feelings of burnout and isolation both for oncologists [29] and oncology nurses [30].

Reported experiences of oncology staff reflect wider systemic concerns, as reported in the Royal Commission into Victoria's Mental Health System [31]. The report highlighted the inequity of service access and the need to provide care that is accessible and integrated into current care pathways both in hospitals and in the community. The heightened emphasis on mental health due to the Royal Commission and COVID lockdowns has spotlighted limited mental health resources. Without adequate support for change, the visibility of this service gap may demoralize staff over time, eroding the coherent desire for change observed in this study. Organisational and government support is necessary not only to improve service accessibility for patients, but also to support HCPs to address patient mental health. While health and quality of life benefits of mental health support for people with cancer are known [32, 33] and previous research has determined psychological interventions are economically beneficial [34], updated economic modelling quantifying the potential financial benefit to institutions and the wider community may assist with funding mental health support in oncology.

### 4.1 Study Limitations

There is a potential bias among participants, as those who chose to participate in the focus groups may have particularly strong views or experiences related to mental health care in oncology, which may not be representative of all staff. Another limitation is that the application of NPT focuses primarily on understanding healthcare practices from the perspective of healthcare professionals, thereby neglecting the patient perspective. Confirming and complementing the proposed changes with direct patient feedback would assist with confirming proposed changes effectively meet patient needs. Future research should aim to incorporate patient perspectives to provide a more comprehensive understanding of integration of mental health support within oncology care. Group sizes ranged from 2 to 5 participants. While smaller groups may yield less interactive discussion, it was an active choice made to reduce potential power dynamics where staff were in groups with their own managers. Future studies could consider grouping participants across different institutions to further reduce these dynamics and foster broader exchange of ideas and perspectives. As this was a single institution study, further research at other institutions, particularly those with dedicated psychological support for oncology patients may help to contrast and confirm perspectives and experiences across the healthcare system. Furthermore, documenting perspectives of other job roles not represented at this institution (e.g. in community health) may be helpful to further understanding system‐wide concerns, particularly for allied health staff. Including the perspectives of mental health clinicians working in oncology departments would also strengthen results, especially for those institutions where such staff are already integrated. Similarly, as this study was conducted within the Australian healthcare system, findings may not reflect experiences of oncology staff working internationally due to differences in healthcare systems, funding and the degree of mental health integration.

### 4.2 Clinical Implications

In the long term, hiring specialist mental health staff and integrating them into oncology departments will likely have the greatest impact on enhancing the ability of oncology staff to support patients' mental health during treatment, and ultimately will benefit patients who will then have access to more comprehensive mental health support. This study highlighted several constraints that are impeding the ability of staff to engage in mental health assessment and management as part of their normal workflow despite the expressed desire to improve current practices, and provides direction for shorter term, impactful changes. Combatting feelings of futility and low efficacy among staff may provide the most impactful changes in the short‐term with minimal budget required. Organisational support for more open conversations around mental health for example through formal multidisciplinary peer support would allow staff to take advantage of vast experiential learning present within teams as well as providing opportunities to undermine feelings of futility expressed in this study.

## 5 Conclusion

The need to improve the management of mental health in oncology outpatient settings is evident. Broader systemic constraints including lack of time, space and training to facilitate discussions regarding patient mental health may make rapid, large‐scale changes challenging. However, this study demonstrates that short‐term initiatives such as more explicit documentation of mental health concerns in medical records, creation of central referral pathways, and clinical supervision for all staff about patient mental health can help and were reported as desirable by oncology staff.

## Author Contributions

**Elizabeth Matthews:** conceptualization, data curation, investigation, formal analysis, methodology, project administration, writing – original draft. **Kate Webber:** conceptualization, validation, writing – review and editing. **Joshua F. Wiley:** conceptualization, validation, writing – review and editing. **Catriona Parker:** conceptualization, investigation, formal analysis, methodology, validation, writing – review and editing.

## Conflicts of Interest

The authors declare no conflicts of interest.

## Supporting information

Supporting Information S1

## Data Availability

The data that support the findings of this study are available from the corresponding author upon reasonable request.
