# Supplementary material for: Providing Outpatient Oncology Mental Health Support: Understanding Staff Perspectives
Source: Psychooncology. 2025 Jun 13;34(6):e70206. doi: 10.1002/pon.70206 (PMC12166112; doi:10.1002/pon.70206)
Supplement: Supplementary file 1 — Supporting Information S1 [file PON-34-e70206-s001.docx]

Supplementary Materials: Consolidated criteria for reporting qualitative studies (COREQ): 32-item checklist

*Based on: Tong A, Sainsbury P, Craig J. Consolidated criteria for reporting qualitative research (COREQ): a 32-item checklist for interviews and focus groups. Int J Qual Health Care. 2007;19(6):349-57.*

| **No. Item** | **Guide questions/description** | **Answer** |
| --- | --- | --- |
| **Domain 1: Research team and reﬂexivity** | | |
| *Personal Characteristics* | | |
| 1. Interviewer/facilitator | Which author/s conducted the interview or focus group? | E Matthews and C Parker facilitated all focus groups, E Matthews led groups with input from C Parker as needed |
| 2. Credentials | What were the researcher’s credentials? E.g. PhD, MD | E Matthews is a PhD candidate. C Parker, K Webber and J Wiley all hold PhD’s, K Webber additionally is an MD |
| 3. Occupation | What was their occupation at the time of the study? | E Matthews: PhD candidate (Clinical Psychology)  C Parker: research fellow  K Webber: medical oncologist/researcher  J Wiley: research fellow |
| 4. Gender | Was the researcher male or female? | E Matthews, C Parker and K Webber are all female. J Wiley is male |
| 5. Experience and training | What experience or training did the researcher have? | All interviewers were trained on the focus group guide and had prior experience completing qualitative interviews |
| *Relationship with participants* | | |
| 6. Relationship established | Was a relationship established prior to study commencement? | E Matthews: Holds administrative role in department where all participants work, known to most participants  C Parker: None before or after interviews  K Webber: Employed in workplace of interest, involvement in research project known to participants but was only involved in analysis of de-identified transcripts  J Wiley: Known to some participants through research networks, involvement in research project known to participants but was only involved in analysis of de-identified transcripts |
| 7. Participant knowledge of the interviewer | What did the participants know about the researcher? e.g. personal goals, reasons for doing the research | Participants were aware of the researchers’ professional affiliations and the purpose of the research, as well as personal motivations of E Matthews related to using the research results to obtain a PhD |
| 8. Interviewer characteristics | What characteristics were reported about the interviewer/facilitator? e.g. Bias, assumptions, reasons and interests in the research topic | Researchers all have an interest in the research topic |
| **Domain 2: study design** | | |
| *Theoretical framework* | | |
| 9. Methodological orientation and Theory | What methodological orientation was stated to underpin the study? e.g. grounded theory, discourse analysis, ethnography, phenomenology, content analysis | Thematic content analysis was used to analyze data with themes then organized against the framework of normalization process theory |
| *Participant selection* | | |
| 10. Sampling | How were participants selected? e.g. purposive, convenience, consecutive, snowball | Purposive and snowball sampling techniques via pre-existing hospital staff networks were used for recruitment |
| 11. Method of approach | How were participants approached? e.g. face-to-face, telephone, mail, email | Face-to-face and by email |
| 12. Sample size | How many participants were in the study? | 26 |
| 13. Non-participation | How many people refused to participate or dropped out? Reasons? | All oncologists and all nurses working in the chemotherapy day unit were offered the chance to participate.  3 participants who expressed interest were unable to be scheduled to a focus group. |
| *Setting* | | |
| 14. Setting of data collection | Where was the data collected? e.g. home, clinic, workplace | All focus groups were completed via Zoom, participants were able to choose the setting they participated from |
| 15. Presence of non-participants | Was anyone else present besides the participants and researchers? | No |
| 16. Description of sample | What are the important characteristics of the sample? e.g. demographic data, date | Please see Table 1 in paper |
| *Data collection* | | |
| 17. Interview guide | Were questions, prompts, guides provided by the authors? Was it pilot tested? | The focus group guide was developed based on the wider literature and in consultation with all members of the research team, it was not pilot tested but was reviewed by K Webber who is a member of the population of interest |
| 18. Repeat interviews | Were repeat interviews carried out? If yes, how many? | No |
| 19. Audio/visual recording | Did the research use audio or visual recording to collect the data? | All focus groups were both video and audio recorded via Zoom |
| 20. Field notes | Were ﬁeld notes made during and/or after the interview or focus group? | Yes – E Matthews field notes during the focus groups |
| 21. Duration | What was the duration of the interviews or focus group? | *Mean* = 58 min; range 55–62 minutes  Focus group 1: 60 minutes  Focus group 2: 55 minutes  Focus group 3: 60 minutes  Focus group 4: 60 minutes  Focus group 5: 60 minutes  Focus group 6: 55 minutes  Focus group 7: 50 minutes  Focus group 8: 62 minutes |
| 22. Data saturation | Was data saturation discussed? | Yes, discussed amongst research team prior to ceasing recruitment and data collection |
| 23. Transcripts returned | Were transcripts returned to participants for comment and/or correction? | No |
| **Domain 3: analysis and ﬁndings** | | |
| *Data analysis* | | |
| 24. Number of data coders | How many data coders coded the data? | E Matthews independently coded the data and discussed coding decisions with the rest of the research team throughout the coding and re-coding process |
| 25. Description of the coding tree | Did authors provide a description of the coding tree? | Organization of open codes into axial codes and themes is referenced in methods of paper, coding tree is included below to demonstrate organization  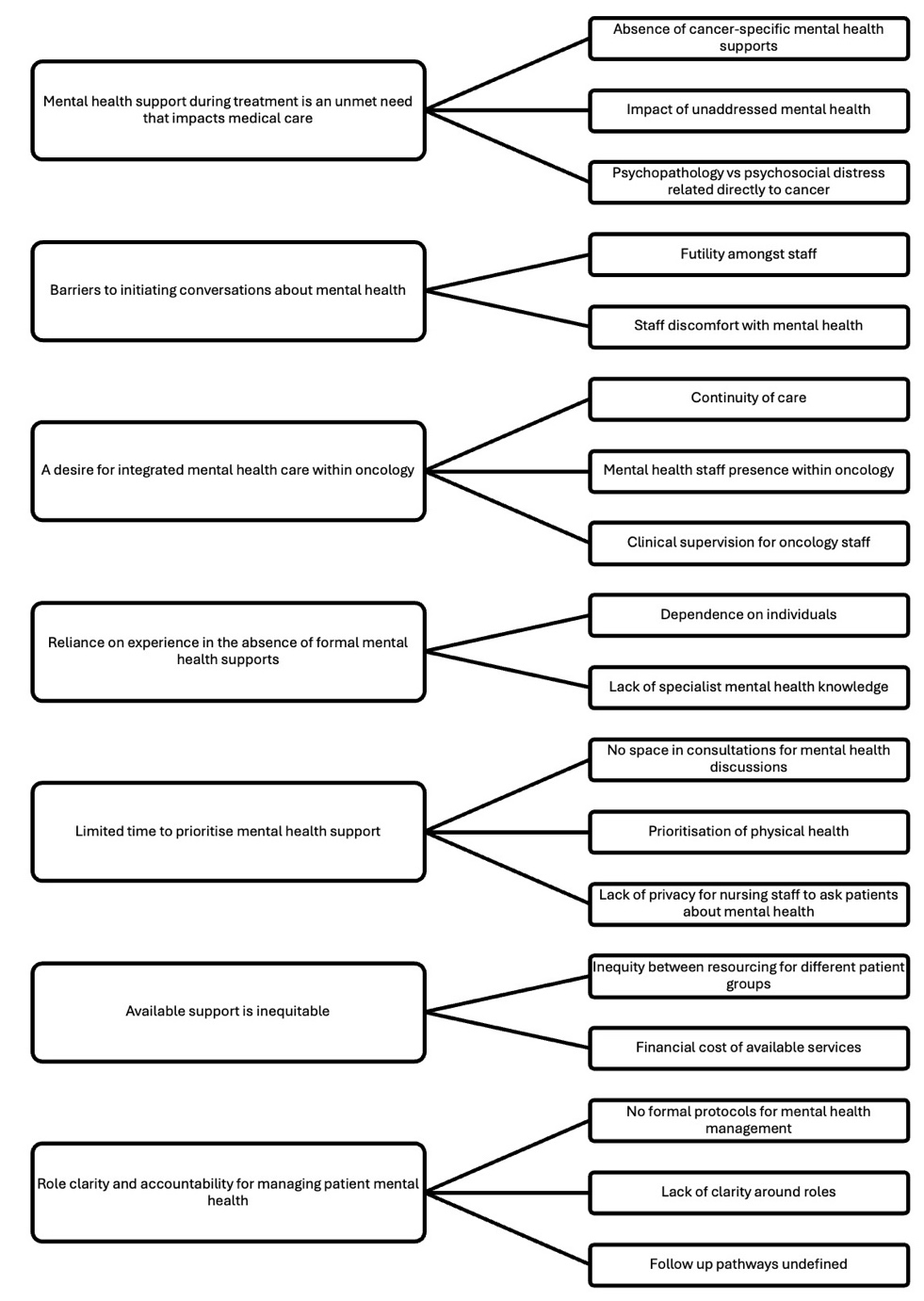 |
| 26. Derivation of themes | Were themes identiﬁed in advance or derived from the data? | Themes were derived from the data |
| 27. Software | What software, if applicable, was used to manage the data? | *NVivo* (Version 14.23.4, QSR International) was used to facilitate coding and analysis. |
| 28. Participant checking | Did participants provide feedback on the ﬁndings? | No |
| *Reporting* | | |
| 29. Quotations presented | Were participant quotations presented to illustrate the themes/ﬁndings? Was each quotation identiﬁed? e.g. participant number | Yes, quotes are all presented with participant number |
| 30. Data and ﬁndings consistent | Was there consistency between the data presented and the ﬁndings? | The research team has made every effort to ensure reported results accurately reflect data gathered |
| 31. Clarity of major themes | Were major themes clearly presented in the ﬁndings? | Major themes are discussed in our Results and Discussion sections |
| 32. Clarity of minor themes | Is there a description of diverse cases or discussion of minor themes? | Minor themes were not presented, as they were not directly relevant to the study aims or broadly transferable beyond this specific site. They specifically included reflections on local referral pathways, changing to site staffing over time, and inpatient care models. This site-specific content was shared informally with the participating institution to support future service development. |
